# A theoretical study on predicted protein targets of apple polyphenols and possible mechanisms of chemoprevention in colorectal cancer

**DOI:** 10.1038/srep32516

**Published:** 2016-09-02

**Authors:** Bernardina Scafuri, Anna Marabotti, Virginia Carbone, Paola Minasi, Serena Dotolo, Angelo Facchiano

**Affiliations:** 1 CNR-ISA, National Research Council, Institute of Food Science, Avellino, Italy; 2 Department of Chemistry and Biology “A. Zambelli”, University of Salerno, Fisciano (SA), Italy

## Abstract

We investigated the potential role of apple phenolic compounds in human pathologies by integrating chemical characterization of phenolic compounds in three apple varieties, computational approaches to identify potential protein targets of the compounds, bioinformatics analyses on data from public archive of gene expression data, and functional analyses to hypothesize the effects of the selected compounds in molecular pathways. Starting by the analytic characterization of phenolic compounds in three apple varieties, i.e. Annurca, Red Delicious, and Golden Delicious, we used computational approaches to verify by reverse docking the potential protein targets of the identified compounds. Direct docking validation of the potential protein-ligand interactions has generated a short list of human proteins potentially bound by the apple phenolic compounds. By considering the known chemo-preventive role of apple antioxidants’ extracts against some human pathologies, we performed a functional analysis by comparison with experimental gene expression data and interaction networks, obtained from public repositories. The results suggest the hypothesis that chemo-preventive effects of apple extracts in human pathologies, in particular for colorectal cancer, may be the interference with the activity of nucleotide metabolism and methylation enzymes, similarly to some classes of anticancer drugs.

Many studies investigate the effects of food antioxidants in human health. The chemo-preventive activity against pathologies as cancer and cardiovascular diseases has been ascribed to their ability of deactivating free radicals. Molecular mechanisms seem to involve the interaction of antioxidants with cellular signaling pathways that mediate cell function, under both normal and pathological conditions. Several studies show an involvement of polyphenols in the MAP kinase (ERK, JNK, p38) pathway and PI3 kinase/Akt signaling cascades, consequently influencing the cellular processes involved in the initiation and progression of cancer and neurodegeneration^1^. Certain polyphenols exert strong inhibitory effects on the growth of colon carcinoma cell through the inhibition of p38/CREB signaling, a decrease of COX-2 expression and stimulation of a G2/M phase cell cycle block; COX-2 gene expression is kept under control by signalling through a number of pathways, including the mitogen-actived protein kinase (MAPK) pathway^2^. The search for protein targets of antioxidants revealed that these compounds might also act by interfering directly on the protein activities. Unfortunately, few studies in the literature have been focused to the binding between antioxidant molecules and specific protein targets As an example, the interaction of two antioxidants (i.e., 1, 2, 8-trihydroxy-6-methoxyxanthone and 1, 2-dihydroxy-6-methoxyxanthone-8-O-β-d-xylopyranosyl) with glucokinase, fructose-1,6-bisphosphatase 1, 11-β-hydroxysteroid dehydrogenase, and modeled protein sulfonylurea receptor has been investigated by *in vivo* and *in silico* assays^3^.

Among food categories rich in antioxidant components, apple fruits seem particularly interesting, both for the number of varieties and their high content of antioxidants^4^,^5^,^6^, and for their known chemo-preventive effects against colorectal cancer^7^,^8^,^9^,^10^. Recently, the content of phenolic compounds in different apple varieties has been compared^11^. Despite the variability of components and their content in the different species, it emerges that apple varieties are characterized by a high content of antioxidants and offer potential benefits for human health. Among the different varieties, some studies focused their attention to Annurca apple fruit, one of the most important cultivars of Southern Italy^12^ and investigated Annurca apple peel content of polyphenols, its antiproliferative effects^13^, its higher ability of reducing cell cholesterol uptake in comparison to other species^14^ and its possible activity in chemoprevention of colorectal cancers^15^,^16^. It has been reported that Annurca apple displays stronger antioxidant activity than other apple varieties^17^, so this evidence attracts more investigations on this variety.

In this article, we describe our work based on the integration of: i) analytic approaches to characterize in detail the content of phenolic compounds in the Annurca and in other two very common apple varieties, namely Red Delicious and Golden Delicious; ii) computational approaches to verify by reverse docking the potential protein targets of these phenolic compounds; iii) direct docking validation of the potential protein-ligand interactions with selected targets; iv) comparison with experimental high-throughput studies and investigations of the biological networks that involve the best protein candidates to be interactors of apple polyphenols, by bioinformatics tools. Results suggest possible molecular mechanisms at the basis of chemo-preventive effects of apple polyphenolic compounds in human pathologies.

## Results and Discussion

### Identification and quantification of polyphenols in the apple extracts

**Figure 1:**
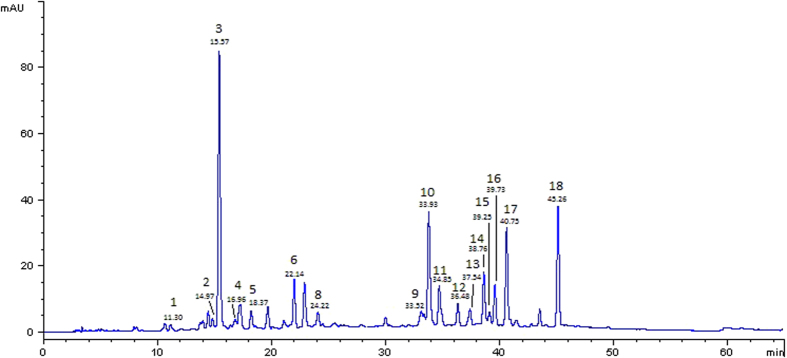
HPLC chromatogram of Annurca apple whole fruit extract, recorded at 280 nm. Peaks are labeled according to Table 1.

Apple extracts were prepared from three different varieties, Annurca, Red Delicious and Golden Delicious, and their total polyphenolic contents were measured by Folin–Ciocalteau’s method. Among the polyphenolic extracts coming from different varieties of apples tested, the one from Red Delicious apple exhibited the highest total phenol content (Table 1). The chemical differences in polyphenols content between the different apple varieties were finely achieved through the analysis and characterization of the three extracts by HPLC-UV/Vis and ESI–ITMS^n^. Identification was achieved on the basis of pseudomolecular [M-H]^−^ (or [M]^+^ for cyanidin-3- *O*-galactoside) ions, together with the interpretation of their collision induced dissociation (CID) fragments. When authentic standards were available, identification was conducted by comparing retention times and MS^n^ fragmentation spectra with those of standards. Nineteen compounds were characterized in the three apple extracts and the classes of polyphenols detected were in agreement with those already reported in previous studies on these or other different apple cultivars, performed on peels only^11^,^14^,^18^. In particular, ESI-ITMS^n^ identification of individual phenolic compounds in the different extracts confirmed the presence of derivatives of the hydroxycinnamic acids (chlorogenic acid and p-coumaroylquinic acid), flavanols (catechin, epicatechin), procyanidins (procyanidins B1, B2 and trimers), flavonols (quercetin-3-*O*-rutinoside, -3-*O*-galactoside, -3-*O*-glucoside, -3-*O*-xyloside, -3-O-arabinopyranoside, -3-*O*-arabinofuranoside, -3-*O*-rhamnoside and -*O*-pentoside), dihydrochalcones (phloretin-2-*O*-xyloglucoside and phloridzin) and anthocyanins (cyanidin-3-*O*-galactoside) in all the three different apple varieties. As an example, the HPLC chromatogram of Annurca apple whole fruit extract, recorded at 280 nm is shown in Fig. 1 and the list of compounds identified in this extract is reported in Table 1. Total and individual polyphenol content of the three different apple varieties is summarized in Table 2.

### Reverse docking results

For each antioxidant investigated (whose chemical structures are reported in Supplementary Table 1), we performed a large-scale screening for protein targets by using idTarget. The search returned a list with thousands of possible protein targets identified among the structures available in PDB. Since we were interested to the effects of antioxidant molecules on human being, only the best human protein targets directly retrieved by idTarget or homologous of a selected target protein and with high structural reliability were taken into account by applying the selection protocol described in Methods.

At the end of this selection process, for each antioxidant we obtained at least two human protein targets, which are listed in Table 3. At a first sight, it appears evident that several antioxidants, despite their different chemical structure, have many protein targets in common, suggesting that only few molecular pathways are targeted by these molecules. Moreover, in some cases, the targets retrieved by a single antioxidant molecule share similar functions, indicating that some antioxidants might be particularly selective for a specific metabolic pathway. For example, all targets obtained for [+]-catechin are Ras-related proteins.

### Docking studies

In order to analyze directly the molecular interactions between the chemical and the selected human protein targets, a docking approach was applied to each target of Table 3. The complete results of this step are shown in Supplementary Table 2.

**Figure 2:**
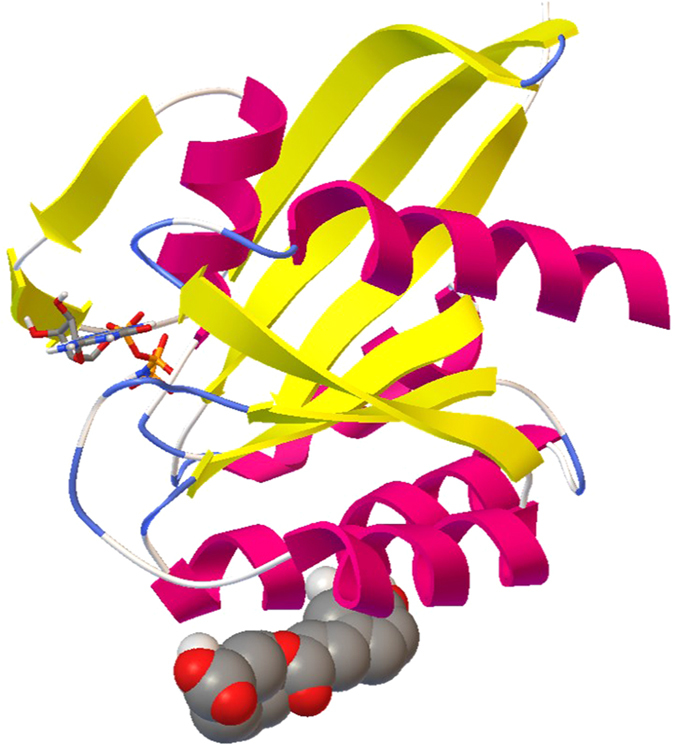
Picture of the best result (run 16) of the blind docking of chlorogenic acid towards GTPase H-Ras. Chlorogenic acid (represented in CPK mode) binds to a cavity different with respect to the active site containing GTP (represented in ball and stick mode). Secondary structures of the protein are shown and colored magenta for alpha helices, yellow for beta sheets and blue for turns. Atoms of the ligands are in the following color code: carbon: gray, oxygen: red, nitrogen: blue, hydrogen: white, phosphorus: orange. The picture has been created using AutoDockTools.

First, a blind docking approach was applied in order to allow each antioxidant to find a possible binding site independently from the ligand binding site known for each protein. In most cases, we found that the selected antioxidant can bind also cavities different from the active site, sometimes with a notable binding affinity. For example, chlorogenic acid appears to bind to GTPase H-ras (PDB code: 3K8Y) also in a cavity different from the binding site, with a predicted binding energy of −11.65 Kcal/mol, corresponding to a predicted Ki in the low nanomolar range (Fig. 2). In general, however, the binding to these alternative pockets is characterized by a lower affinity with respect to the “canonical” binding site. An exception is the interaction between cyanidin-3-galactoside and the proteins GTP-binding nuclear protein Ran (PDB code: 2MMC) and GTP-ase Kras (PDB code: 4OBE). Indeed, in both cases the interaction in a binding site alternative to the canonical one has a predicted binding energy better than the one associated to the interaction identified with a docking focused on the binding site of the protein (Supplementary Table 2). In this last case, however, despite its position, the ligand does not interact with residues belonging to the active site of the protein.

The docking of the antioxidants focused in the known binding site of the proteins was performed both in the absence and in the presence of ions/cofactors when they are present, to compare the affinity of the antioxidant molecule to the protein target in both conditions and to understand if the antioxidant was bound to a functionally active molecule. Results are shown in Supplementary Table 2.

**Figure 3:**
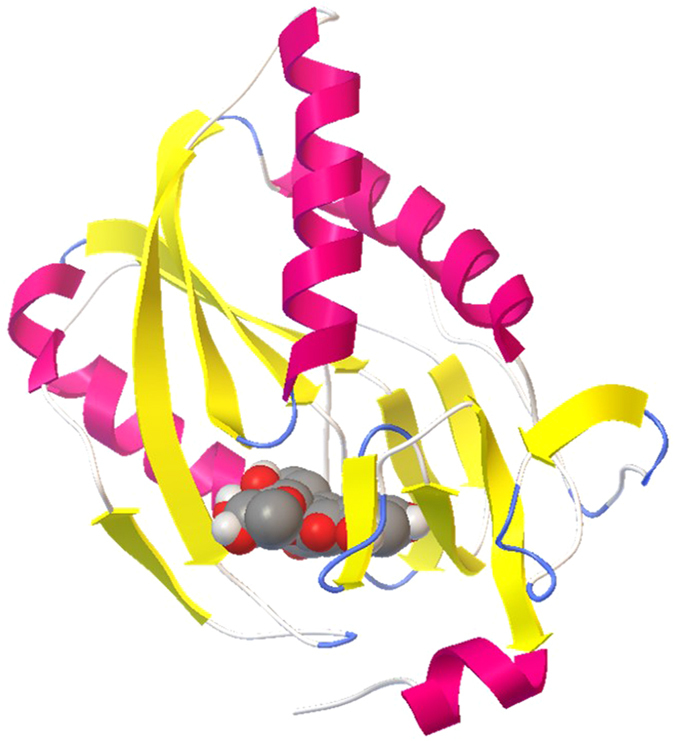
Picture of the best result (run 40) of the docking of quercitrin focused on the binding site of hypoxanthine-guanine phosphoribosyltransferase. Representations and color codes are as in Fig. 2.

Some proteins selected by idTarget do not bind any cofactor. In these cases, the predicted binding energies of the various antioxidant molecules toward their predicted targets vary between −11.22 and −3.08 kcal/mol. The lowest (better) value corresponds to the interaction of quercitrin with Hypoxanthine-guanine phosphoribosyltransferase (PDB code: 1BZY) (Fig. 3); the highest (worse) one to the interaction of procyanidin B1 with the light chain of factor X (PDB code: 3KL6). Other interactions with a predicted binding energy lower than −10 kcal/mol are those between Hypoxanthine-guanine phosphoribosyltransferase and avicularin (−11.06 kcal/mol); between matrix metalloproteinase 16 (PDB code: 1RM8) and [+]-epicatechin (−10.58 kcal/mol); between Guanidinoacetate N-methyltransferase (PDB code: 3ORH) and hyperin (−10.30 kcal/mol), or isoquercitrin (−10.88 kcal/mol), or phloridzin (−10.01 kcal/mol) or rutin (−11.03 kcal/mol); finally, between Heparan sulfate glucosamine 3-O-sulfotransferase 1 (PDB code: 1ZRH) and phloridzin (−10.27 kcal/mol).

**Figure 4:**
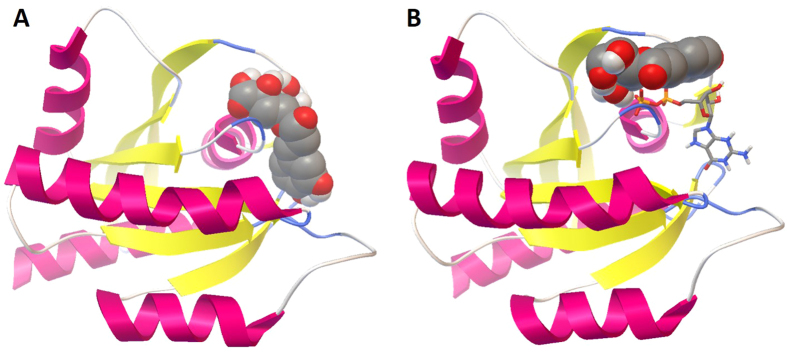
Picture of the best result of the docking of chlorogenic acid focused on the binding site of GTPase Kras, in the absence (**A**) or in the presence (**B**) of the cofactor GTP. Representations and color codes are as in Fig. 2.

**Figure 5:**
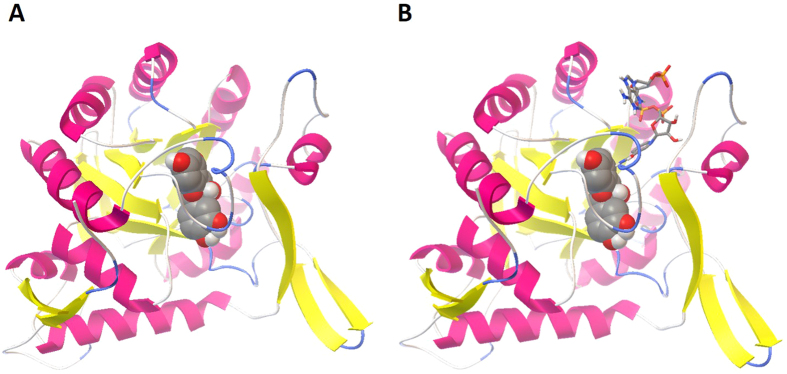
Picture of the best results of the docking of [+]-epicatechin focused on the binding site of GMP reductase, in the absence (**A**) or in the presence (**B**) of the cofactor NADPH. Representations and color codes are as in Fig. 2.

When the target proteins selected by idTarget have a cofactor bound into the active site, it is possible to note that most antioxidant molecules bind to them with a high affinity only when cofactors are not included in the binding pocket. For example, chlorogenic acid can interact with the binding sites of GTPase Kras (PDB code: 4OBE) with a predicted affinity in the low nanomolar range, only in the absence of the cofactor, whereas in the presence of GTP the predicted binding energy is much higher. Indeed, when GTP is not bound to the protein, the molecule is able to fit perfectly in the active site, occupying the same position of the cofactor (Fig. 4A), but when GTP is bound to the active site, due mainly to the steric hindrance of the cofactor, the antioxidant is forced to assume another position, with a less favorable binding energy (Fig. 4B). In other cases, instead, the binding to the active site of the protein is possible also in the presence of cofactors, and even favored by them. For example, in the case of the interaction between [+]-epicatechin and GMP reductase 2 (PDB code: 2C6Q), the presence of NADPH not only allows the binding of the antioxidant to the protein in the same way as it happens in the absence of the cofactor (Fig. 5), but the affinity in the first case is slightly higher, suggesting that the cofactor could in its turn interact with the antioxidant (see Supplementary Table 2).

In order to evaluate if the different antioxidants show an affinity for these protein targets comparable to that of their cofactors, we also performed the docking of cofactors into the structures of their proteins, when applicable. Results are shown in Supplementary Table 2. The correct self-docking of the cofactor, reproducing the crystallographic structure, in some cases was particularly hard to obtain. This is generally due to the dimensions of the cofactor and to the high number of rotatable bonds to take into account. Additionally, in the case of GDP bound to protein GTP-ase Kras (PDB code: 4OBE), the water molecules present in the binding site were left in the structure to allow the correct interaction of the cofactor with the protein. Anyway, generally the cofactor interacts with the binding site keeping the same conformation identified in the crystallographic structure. The predicted binding energies of the cofactors to their proteins are often lower than those of the antioxidants (Supplementary Table 2). This suggests that these last molecules, although potentially able to bind to the proteins, are unfavored in the competition with respect to the cofactor. In some cases, however, the predicted binding energies are comparable or even lower. For example, the Ras-related protein Rap 1A (PDB code: 1C1Y) with residues in conformation B binds to [+]-catechin with a predicted binding energy of −8.48 kcal/mol for the result with best energy, and the predicted binding energy of the cofactor GTP is −9.98 kcal/mol. The same for cyanidine-3-galactoside: in these two cases, indeed, the two antioxidants seem to interact better with the residues of the binding site in the alternative conformation B, whereas the GTP interacts strongly with the residues of the binding site in the alternative conformation A. Other interactions of the antioxidants with predicted binding energies similar to that of the cofactor are the one of reynoutrin with prostaglandin reductase 2 (PDB code: 2ZB4), and the one of rutin with amine oxidase B (PDB code: 2BK3), ornithine aminotransferase (PDB code: 2OAT) and prostaglandin reductase 2.

In order to select the most probable interactions of antioxidants with protein targets for further studies, we decided to apply cut-offs to binding energies and the number of poses in the cluster. In particular, we selected for further studies those proteins with predicted binding energies with the antioxidants lower than −7 kcal/mol and a number of poses higher than 30, and with a predicted binding energy difference no higher than 1.50 kcal/mol (corresponding to the intrinsic error of the program AutoDock) with respect to the predicted binding energy of the cofactor, if applicable. Table 4 lists the proteins, with the interacting antioxidant(s), further evaluated in the following step of the work.

### Bioinformatics-driven functional analysis and interpretation

The list of 18 potential protein targets of the investigated antioxidants has been analyzed with bioinformatics tools to obtain a functional analysis in terms of gene expression data and interaction networks. First, we focused our attention on experimental data within public repositories, obtained from studies concerning pathologies for which the polyphenolic apple extracts have been reported to have chemopreventive action. Table 5 reports the gene expression of the genes coding for the 18 proteins, extracted by BioGPS resource^19^, in colorectal adenocarcinoma, using data from normal colon cells as control. Some of the gene transcripts of the target proteins are differentially expressed at a significant extent in colorectal cancer cells with respect to control (colon cells). In particular, the most significant differences concern OAT and RAP1A, both down-regulated, and DCXR, GAMT, GMPR2, HPRT1, KDM1A, PRMT3, and HSPA1B, up-regulated in colorectal adenocarcinoma. This analysis suggests that the polyphenol compounds investigated are potentially able to interact with gene products strongly involved in colorectal cancer, and the effects of apple antioxidants against this pathology could be related to their capability to bind these proteins with a possible inhibitory effect.

**Figure 6:**
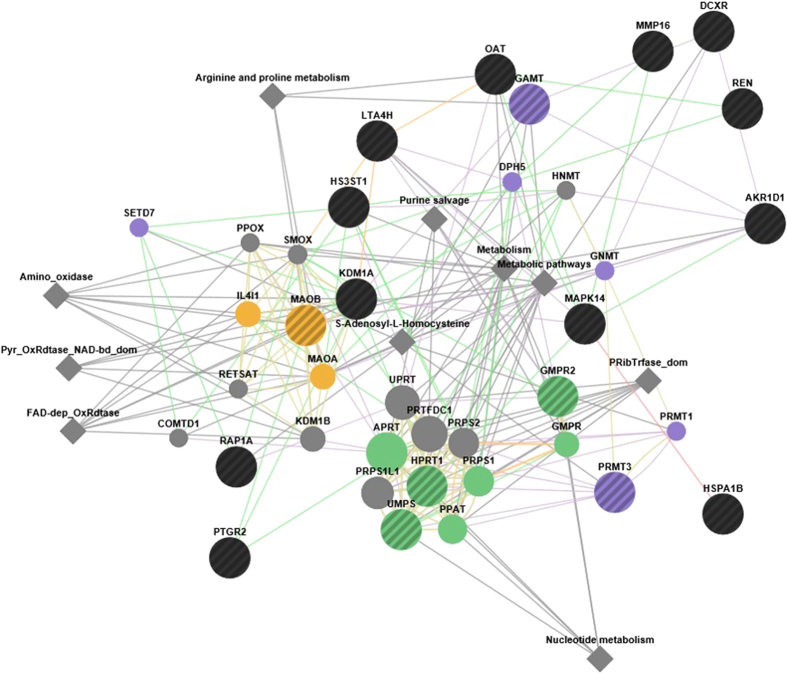
Network of interactions of the 18 potential target proteins, generated with GeneMANIA. The network has been extended to include 20 related genes and at most 10 related attributes. The analyses by including less (i.e 10) or more (i.e. 50) genes modify the complexity of the image but leave unchanged the functional interpretation. Some functional groups of genes are coloured: oxidoreductase activity (yellow), purine nucleobase metabolic process (green), S-adenosylmethionin-dependent methyltransferase activity (purple). Nodes with dashes (both black and coloured ones) indicate the targets of antioxidants. Other functional groups are mainly overlapping with the similar ones (i.e, methyltransferase activity overlaps S-adenosylmethionin-dependent.

Figure 6 shows the network of interactions involving the 18 selected target proteins, obtained from the analysis by GeneMANIA. Table 6 lists the biological functions associated to each protein included in the network. The most significant functions identified involve nucleotide metabolism, methyltransferase activity and oxidoreductase activity. While the latter one is expected for molecules with antioxidant properties, the others represent a very interesting finding, in consideration that a large number of anticancer drugs are targeted to nucleotide metabolism enzymes^20^. Moreover, methylation/demethylation processes are of extreme relevance in gene regulation, and involved in various processes of cancer development and progression^21^, also in colorectal cancer^22^. Indeed epigenetic drugs such as azacytidine, its deoxy derivative 5-aza-2′-deoxycytidine (decitabine) and hydralazine have been developed to act as inhibitors of DNA methyltransferases involved in epigenetic regulation phenomena and are currently used in the treatment of myelodysplastic syndromes, but their severe side effects discourage their use in other tumors^23^.

This work suggests a possible molecular interpretation of the fact that apple antioxidants can exert both a chemopreventive and a chemotherapeutic activity in colorectal cancer. Many example in literature support the chemopreventive activity of apple extracts specifically against colon cancer, either in *in vitro* and *in vivo* models (reviewed in ref. 10). In particular, a study on a mouse model of familial adenomatous polyposis exposed to a high-risk diet *vs* a balanced diet showed that apple polyphenolic extract assumed in doses compatible to the normal daily consumption of apples is able to reduce the risk of developing cancer by protecting against hypomethylation, especially in normal tissues. Notably, this effect is evident not only in individuals eating a high-risk diet, but also in individuals subjected to the balanced diet^15^. Some studies highlighted also the ability of apple polyphenols to act as chemotherapeutic agents: for example, the apple polyphenolic extract has been shown to induce reactivation of DNA repair genes such as hMLH1, p14^ARF^ and p16^INK4a^ via promoter demethylation in an *in vitro* model of colorectal cancer cells, an effect similar to the one exerted by the epigenetic drug decitabine at a concentration of the same order of magnitude of the extract^16^. The low dosage to which the apple extract exerts its effects induces to suppose a synergistic action of its different compounds. Our analysis confirms indeed that different apple polyphenols can target different proteins involved in these regulatory pathways (for example, KDM1A is the preferred target of hyperin whereas GAMT is the target of many compounds such as hyperin, isoquercitrin, phloridzin and rutin) and therefore supports the hypothesis that they can act as a cocktail of compounds able to interact with proteins involved in nucleotide metabolism and/or with methyltransferase enzymes with an effect similar to anticancer drugs.

## Materials and Methods

### Chemicals

Acetone (HPLC grade), 1-Butanol (for liquid chromatography) and Acetonitrile (HPLC grade) were obtained from Merck (Darmstadt, Germany). Glacial acetic acid and sodium carbonate were purchased from Carlo Erba (Rodano, Milan, Italy). Chlorogenic acid, phloridzin (phloretin-2-*O*-glucoside), quercitrin (quercetin-3-*O*-rhamnoside), rutin (quercetin-3-*O*-rutinoside) and Folin–Ciocalteu’s reagent were purchased from Sigma Chemical Company (St. Louis, USA). Procyanidin B2, [−]-epicatechin, isoquercitrin (quercetin-3-*O*-glucoside) were obtained from Fluka (Buchs SG, Switzerlandand). Naringenin was purchased from ChromaDex (Irvine, CA, USA). Cyanidin-3-*O*-galactoside chloride and [+]-catechin were obtained from Extrasynthese (Genay, France). HPLC grade water (18.2 MO) was prepared by using a Millipore Milli-Q purification system (Millipore Corp., Bedford, MA, USA).

### Fruit collection and sample treatment

Five apples of the three varieties Annurca (*Malus pumila* Miller cv Annurca), Red Delicious (*M. pumila* Miller cv Red Delicious), and Golden Delicious (*M. pumila* Miller cv Golden Delicious), were randomly selected from batches purchased on different days in a local supermarket in Avellino (Italy). All fruits were quickly washed in distilled water, cut into quarters, and seeds and core were removed. Fruit parts were finely chopped and, for each variety, aliquots of 1 g were treated with 5 ml of 80% aqueous acetone to extract phenolic compounds. Each extraction was carried out for 24 h on a horizontal shaker in a refrigerated chamber at 4 °C. After centrifugation, the supernatant was removed, the pellet was suspended in 5 ml of 80% aqueous acetone and the extraction was carried out for a further 24 h under the same conditions. The two supernatants were pooled and dried in a rotary evaporator (LaboRota 4000/HB Efficient, Heidolph, Schwabach, Germany). Dry extracts were suspended in a mixture of water/1-butanol (1/1; v/v) and subjected to liquid-liquid extraction to remove water-soluble ingredients such as sugars, inorganic salts. This operation was repeated three times, and n-butanol phases were recovered, pooled, dried in a rotary evaporator and stored at −20 °C until used.

### Analysis of total phenolic content

The amounts of total phenols in apple extracts were determined according to the Folin–Ciocalteu procedure^24^ using gallic acid as a reference standard. Folin–Ciocalteu’s reagent (62.5 μL) and 250 μL of distilled water were added to 62.5 μL of suitable aqueous dilutions of dry extracts. The reaction mixture was mixed and allowed to stand for 6 min. Finally, 625 μL of sodium carbonate and 500 μL of distilled water were added and the solution was incubated in the dark for 90 min. The sample absorbances were measured at 760 nm. The results were expressed as equivalents of gallic acid (GAE), in mg/100 g of fresh weight (FW). All measurements were carried out in triplicate.

### High Performance Liquid Chromatography-Ultraviolet/Visible (HPLC–UV/Vis) analyses

Extracts from the three different apple varieties were reconstituted in 1% formic acid and analyzed by HPLC-UV/Vis on a HP 1110 series HPLC (Agilent, Palo Alto, CA, USA) equipped with a binary pump (G-1312A) and an UV detector (G-1314A) according to procedure previously described^13^. In detail, individual phenols were separated on a Hypersil BDS C18 column (250 mm × 4.6 mm, 5 μm) (Thermo, Bellefonte, PA, USA) at a flow rate of 1 ml min^−1^; solvent A was 2% acetic acid and solvent B was 0.5% acetic acid in acetonitrile and water (50:50, v/v). After a 5 min hold at 10% solvent B, elution was performed according to the following conditions: from 10% (B) to 55% (B) in 50 min and to 95% (B) in 10 min, followed by 5 min of maintenance. Flavonols, procyanidins, dihydrochalcones, flavanols and hydroxycinnamic acids were monitored at 280 nm and anthocyanins at 520 nm. For quantitative analysis, standard curves for each polyphenol standard were prepared over a concentration range of 0.1–1 μg μl^−1^ with six different concentration levels and duplicate injections at each level. Peak area ratios between the areas of each polyphenol standard and those of naringenin (0.3 μg μl^−1^), used as internal standard, were calculated and plotted against the corresponding standard concentration, using weighed linear regression to generate standard curves. Quantification of cyanidin-3-O-galactoside was performed with external calibration curves generated by repeated injections of a fixed volume of standard solutions over a concentration range of 0.01 – 0.1 μg μl^−1^, with five different concentrations and duplicate injections at each level. All samples were prepared and analysed in duplicate. The results were expressed as mg/100 g of fresh weight (FW).

### Electrospray Ionization multistage Ion Trap Mass Spectrometry (ESI-ITMS^n^) analysis

Identification of phenolic compounds present in the different HPLC separated fractions was carried out by ESI-ITMS^n^ using a Finnigan LCQ DECA XP Max ion trap mass spectrometer (Thermo Finnigan, San Josè, CA, USA), equipped with Xcalibur® system manager data acquisition software (Thermo Finnigan, San José, CA, USA). Mass spectra were recorded from mass-to-charge ratio (m/z) 80 to 1800 both in negative and in positive ionization mode. The capillary voltage was set at −28 V, the spray voltage was at 3 kV and the tube lens offset was at −10 V in negative ion mode, while the capillary voltage was set at 34 V, the spray voltage was at 3.5 kV and the tube lens offset was at 55 V in positive ion mode. The capillary temperature was 275 °C. Data were acquired in MS, MS/MS and MS^n^ scanning mode.

### Reverse Docking

In order to identify possible protein targets for several antioxidant molecules extracted from Annurca apple, a reverse docking approach protocol was applied^25^ by using idTarget platform (http://idtarget.rcas.sinica.edu.tw/), a free web-server for the prediction of possible targets for the binding of small chemical molecules through a divide-and-conquer docking approach^26^.

For each antioxidant tested, a file of the 3D molecular structure in .sdf format was downloaded from NCBI-PubChem Compound database (http://pubchem.ncbi.nlm.nih.gov/)^27^ (Supplementary Table 1). Then, these files were converted in the .pdb format using UCSF Chimera (http://www.rbvi.ucsf.edu/chimera/)^28^. The.pdb files were submitted to the idTarget server. For each ligand, idTarget automatically assigned the protonation state; concerning the scanning process, the “fast mode” option was used (the ligand was mapped to the binding site of the homologous proteins structurally aligned and locally minimized by adaptive local searches to remove too close contacts with protein atoms^26^), and the other parameters were set to a default value. Moreover, idTarget removes all ligands/cofactors bound to the proteins before performing the reverse docking procedure.

For each antioxidant molecule, the best twenty unique targets in terms of energy retrieved by idTarget were selected, downloaded from Protein Data Bank - PDB^29^ and analyzed. In most cases, the PDB files identified by idTarget are different structures of the same protein: in these cases, they were counted as a single target (Supplementary Table 2). Only the human protein targets were selected, and the non-human ones were replaced by structurally equivalent human homologues if available, otherwise they were discarded. The structural equivalence was checked by visual inspection and superposition of the two structures. Hypothetical proteins were discarded and mutant proteins were replaced by the equivalent wild type protein, whenever possible, otherwise they were discarded, too, in order to avoid that the presence of the mutation affects the predicted ability of the protein in physiologic conditions to bind the antioxidant. For example, the target identified for Procyanidin B2 with code 3BUR has been replaced with 3BUV, because 3BUR has two mutations, namely Y58F and E120A, which can affect enzyme catalysis^30^. In addition, the resolution of the protein and the correctness of the structure was checked for all selected targets. In order to consider for the following steps only strictly reliable results, those structures at resolution worse than 2.5 Å or with missing residues and atoms were replaced by a complete structure of the same protein in the same 3D conformation, if available, otherwise they were discarded. When structures carry residues in multiple conformations (occupancy <1), each conformation was analyzed separately.

### Molecular docking

To assess the appropriate binding orientations and conformations of the ligand with the proteins selected after this procedure, molecular dockings were performed using AutoDock 4.2^31^. In this study, proteins were kept rigid while the ligands were left flexible. The water molecules were removed from protein structures before calculations, unless otherwise reported (see Results). The ligands and the receptors were prepared using AutoDockTools^31^, adding all hydrogens, Gasteiger charges and atom types. To search for the best conformational space of ligand, the Lamarckian Genetic Algorithm was employed, setting 100 independent Genetic Algorithm runs for each ligand, while other parameters were kept at default value.

In the first step, a “blind” docking was performed setting a grid box that includes the entire protein surface. Furthermore, for proteins formed by two or more identical chains, the blind docking was performed on each single chain, and only the best result among them was chosen. In a following step, a “focused” docking was performed, setting the grid box in order to include only those residues belonging to the binding site of proteins, as it results from the PDB file. Therefore, for each focused docking, both the size and the position of the grid box depend on the size and the position of the binding site in the protein structure. The docking parameters were unchanged from those used for the blind docking. Dockings were made either by keeping the cofactors (identified by annotations in UniProt database^32^) and ions in the receptors’ structures, and removing them. The ligands or substrates bound to the proteins, instead, were always removed from the target’s coordinates. Finally, dockings were also performed between each protein target and its cofactor, whenever present, in order to evaluate the predicted binding energy in comparison to that of the antioxidant bound to the protein. In this case, only a “focused” docking procedure was performed.

The best ligand-protein complexes were identified as those with the lowest binding energy and with the higher number of poses in the cluster.

### Bioinformatics-driven functional analysis

The list of potential targets of apple antioxidants has been investigated by means of bioinformatics resources for functional validation and investigation of their interaction network.

Expression data in colon and colorectal adenocarcinoma for genes coding the potential targets have been extracted by BioGPS^19^, within dataset GeneAtlas U133A. GeneMANIA^33^ has been used for analysis of interaction networks and functional analysis.

## Additional Information

**How to cite this article**: Scafuri, B. *et al*. A theoretical study on predicted protein targets of apple polyphenols and possible mechanisms of chemoprevention in colorectal cancer. *Sci. Rep.* **6**, 32516; doi: 10.1038/srep32516 (2016).

## Supplementary Material

Supplementary Information

## Figures and Tables

**Table 1:** List of compounds identified in the three different apple varieties extracts including quasi-molecular ions and fragment ions.

peak	t_r_ (min) ANNURCA	t_r_ (min) RED	t_r_ (min) GOLDEN	[M-H]^−^ *m/z*	MS/MS ions *m/z*	Identification
1	11,30	10,44		577	451, 425, 407, 289	Procyanidin B1
2	14,97			289	245, 205, 203, 179, 137, 125	[+]-Catechin
3	15,57	14,85	16,00	353	191	Chlorogenic acid
4	16,96	16,13	17,49	865	847, 739, 695, 577, 451, 407, 289	Procyanidin trimer
5	18,37	17,07	18,57	577	451, 425, 407, 289	Procyanidin B2
6	22,14	20,75	22,40	289	245, 205, 203, 179, 137, 125	[−]-Epicatechin
7		21.31	22,81	337	173, 163, 155	p-Coumaroylquinic acid
8	24.09	28,62		449 [M]^+^	287	Cyanidin-3-*O*-galactoside
9	24,22	22,67	24,37	865	847, 739, 695, 577, 451, 407, 289	Procyanidin trimer (isomer)
10	33,52			609	301	Rutin (quercetin-3-*O*-rutinoside)
11	33,93	32,90	34,84	463	301	Hyperin (quercetin-3-*O*-galactoside)
12	34,85	33,83	35,71	463	301	Isoquercitrin (quercetin-3-*O*-glucoside)
13	36,48	35,33	37,23	433	301	Reynoutrin (quercetin-3-*O*-xyloside)
14	37,54	36,59	38,59	433	301	Guajaverin (quercetin 3-*O*-arabinopyranoside)
15	38,76	37,49	39,47	433	301	Avicularin (quercetin 3-*O*-arabinofuranoside)
16	39,25	38,08	40,00	433	301	Quercetin-*O*-pentoside
17	39,73	38,55	40,34	447	301	Quercitrin (quercetin-3-*O*-rhamnoside)
18	40,75	39,49	41,20	567	273	Phloretin-2-*O*-xyloglucoside
19	45,26	42,70	44.61	435	273	Phlorizin (phloretin-2-*O*-glucoside)

**Table 2:** Concentration of individual and total polyphenolics determined by HPLC (mg/100 g of FW) and F-C Method (mg_GAE_/100 g of FW) extracted from Annurca, Red Delicious and Golden Delicious) apples whole fruits.

	Annurca	Red Delicious	Golden Delicious
**Total polyphenols (by Folin-Ciocalteu’s method) GAE**	**25,37 ± 0,33**	**43,10 ± 2,88**	**24,17 ± 0,48**
Chlorogenic acid	4.49 ± 0.01	2,17 ± 0,08	3,23 ± 0,09
p-Coumaroylquinic acid	ND	2,16 ± 0,20	1,51 ± 0,05
**Total hydroxycinnamic acids**	**4.49 ± 0.01**	**4.53 ± 0.28**	**4.74 ± 0.13**
[+]-Catechin	0.60 ± 0.02	ND	ND
[−]-Epicatechin	1.24 ± 0.03	2,37 ± 0,16	1,13 ± 0,06
**Total flavanols**	**1.84 ± 0.04**	**2,37 ± 0,16**	**1,13 ± 0,06**
Procyanidin B_1_	0.35 ± 0.01	0,84 ± 0,01	ND
Procyanidin trimer	0.64 ± 0.01	1,23 ± 0,15	0,86 ± 0,06
Procianidin B_2_	0.89 ± 0.01	2,72 ± 0,30	1,37 ± 0,07
Procyanidin trimer (isomer)	1.01 ± 0.01	1,57 ± 0,22	1,01 ± 0,14
**Total procyanidins**	**2.88 ± 0.04**	**6.37 ± 0.67**	**3.24 ± 0.27**
Cyanidin-3-*O*-galactoside	0.02 ± 0.01	0,17 ± 0.01	ND
**Total anthocyanins**	**0.02 ± 0.01**	**0,17 ± 0.01**	
Rutin (Quercetin-3-*O*-rutinoside)	0.40 ± 0.01	ND	ND
Hyperin (Quercetin-3-*O*-galactoside)	4.45 ± 0.02	7,65 ± 0,46	2,44 ± 0,12
Isoquercitrin (Quercetin-3-*O*-glucoside)	1.76 ± 0.01	1,85 ± 0,17	0,87 ± 0,10
Reynoutrin (Quercetin-3-*O*-xyloside)	1.02 ± 0.09	2,83 ± 0,10	0,79 ± 0,09
Guajaverin (Quercetin 3-*O*-arabinopyranoside)	0.87 ± 0.01	2,66 ± 0,11	0,87 ± 0,11
Avicularin (Quercetin 3-*O*-arabinofuranoside)	1.98 ± 0.01	4,62 ± 0,59	1,37 ± 0,09
Quercetin-*O*-pentoside	0.61 ± 0.01	0,79 ± 0,08	0,24 ± 0,04
Quercitrin (Quercetin-3-*O*-rhamnoside)	1.17 ± 0.01	2,40 ± 0,38	2,58 ± 0,12
**Total flavonols**	**12.27 ± 0.04**	**22.81 ± 1.88**	**9.16 ± 0.67**
Phloretin-2-*O*-xyloglucoside	1.35 ± 0.01	0,29 ± 0,08	0,13 ± 0,03
Phloridzin (phloretin-2-*O*-glucoside)	1.51 ± 0.01	3,47 ± 0,18	1,11 ± 0,07
**Total dihydrochalcones**	**2.86 ± 0.01**	**3.75 ± 0.26**	**1.24 ± 0.10**
**Total polyphenols (by HPLC method)**	**24.37 ± 0.05**	**39,81 ± 3,25**	**19,50 ± 1,23**

Results were expressed as average (mean) concentration ± SD of triplicate (F-C Method) or duplicate (HPLC method).

ND: not detected.

**Table 3:** List of human protein targets for each antioxidant selected for direct docking studies.

Antioxidant	Protein	PDB code
Avicularin	Hypoxantine-guanine phosphoribosyltransferase	1BZY
	Uridine 5′-monophosphate synthase	2QCG
[+]-Catechin	Ras Related protein Rap 1A	1C1Y
	GTP-binding nuclear protein Ran	2MMC
	GTP-ase Hras	3K8Y
	GTP-ase Kras	4OBE
Cyanidin-3-galactoside	Ras Related protein Rap 1A	1C1Y
	ADP-ribosylation factor-like 10B	1ZD9
	Isopentenyl-diphoshate delta isomerase	2ICK
	GTP-binding nuclear protein Ran	2MMC
	Hydroxyacid oxidase 1	2RDU
	GTP-ase Kras	4OBE
[−]-Epicatechin	Matrix metalloproteinase 16	1RM8
	GMP reductase 2	2C6Q
	Uridine 5′-monophosphate synthase	2QCG
	Sulfotransferase family cytosolic 1B member 1	2Z5F
Hyperin	Lysine-specific histone demethylase 1	2IW5
	Ornithine Aminotransferase	2OAT
	Guanidinoacetate N-methytransferase	3ORH
Isoquercitrin	Ornithine Aminotransferase	2OAT
	Guanidinoacetate N-methytransferase	3ORH
Phloridzin	Heparan sulfate glucosamine 3-O-sulfotransferase 1	1ZRH
	Dynamin-1	2X2E
	Heat shock 70kDa protein 1A	3ATV
	L-Xylulose reductase	3D3W
	Guanidinoacetate N-methytransferase	3ORH
Procyanidin B_1_	Renin	2G1Y
	Factor X light chain	3KL6
Procyanidin B_2_	3-oxo-5-beta-steroid 4-dehydrogenase	3BUV
	Leukotriene A-4 hydrolase	3FUN
Quercetin	Mitogen-activated protein kinase 14	1WBS
	Uridine 5′-monophosphate synthase	2QCG
	Aldehyde Reductase	4LB4
Quercitrin	Hypoxantine-guanine phosphoribosyltransferase	1BZY
	Prostaglandin Reductase 2	2ZB4
Reynoutrin	Ornithine Aminotransferase	2OAT
Prostaglandin Reductase 2	2ZB4	
Rutin	Amine Oxidase [Flavin-Containing] B	2BK3
	Arginine N-methyltransferase 3	2FYT
	Lysine-specific histone demethylase 1	2IW5
	Ornithine Aminotransferase	2OAT
	Prostaglandin Reductase 2	2ZB4
	Guanidinoacetate N-methytransferase	3ORH

**Table 4:** List of human protein targets selected for functional analysis.

Protein	PDB CODE	Gene Symbol	Antioxidant
Hypoxantine-guanine phosphoribosyltransferase	1BZY	HPRT1	Avicularin, Quercitrin
Uridine 5′-monophosphate synthase	2QCG	UMPS	Avicularin, [−]-epicatechin, quercetin
Amine Oxidase [Flavin-Containing] B	2BK3	MAOB	Rutin
Ras Related protein Rap 1A	1C1Y	RAP1A	[+]-Catechin, Cyanidin-3-galactoside
Matrix metalloproteinase 16	1RM8	MMP16	[−]-Epicatechin
GMP reductase 2	2C6Q	GMPR2	[−]-Epicatechin
Lysine-specific histone demethylase 1	2IW5	KDM1A	Hyperin
Ornithine Aminotransferase	2OAT	OAT	Isoquercitrin, Reynoutrin, Rutin
Guanidinoacetate N-methytransferase	3ORH	GAMT	Hyperin, Isoquercitrin, Phloridzin, Rutin
Heat shock 70kDa protein 1A	3ATV	HSPA1B	Phloridzin
Heparan sulfate glucosamine 3-O-sulfotransferase 1	1ZRH	HS3ST1	Phloridzin
L-Xylulose reductase	3D3W	DCXR	Phloridzin
Renin	2G1Y	REN	Procyanidin B1
3-oxo-5-beta-steroid 4-dehydrogenase	3BUV	AKR1D1	Procyanidin B2
Leukotriene A-4 hydrolase	3FUN	LTA4H	Procyanidin B2
Mitogen-activated protein kinase 14	1WBS	MAPK14	Quercetin
Prostaglandin Reductase 2	2ZB4	PTGR2	Quercitrin, Reynoutrin, Rutin
Arginine N-methyltransferase 3	2FYT	PRMT3	Rutin

**Table 5:** Gene expression data for the selected antioxidant targets, in colorectal adenocarcinoma and colon as control.

	Colon	Colorectal adenocarcinoma
AKR1D1	7.45 ± 0.53	7.40 ± 1.20
DCXR	133.60 ± 36.13	228.80 ± 49.64
GAMT	9.05 ± 0.74	19.40 ± 2.62
GMPR2	8.80 ± 0.35	18.75 ± 8.95
HPRT1	157.85 ± 41.90	439.05 ± 49.89
HS3ST1	9.85 ± 0.672	9.80 ± 1.56
HSPA1B	245.45 ± 58.73	373.95 ± 143.74
KDM1A	63.90 ± 9.05	83.75 ± 0.39
LTA4H	172.95 ± 19.27	183.25 ± 31.08
MAOB	9.95 ± 0.81	10.55 ± 0.95
MAPK14	5.50 ± 0.424	5.45 ± 0.884
MMP16	6.90 ± 0.07	6.85 ± 0.03
OAT	556.20 ± 1.34	329.55 ± 15.03
PRMT3	8.70 ± 0.63	42.10 ± 1.77
PTGR2	5.60 ± 0.00	5.55 ± 0.03
RAP1A	741.85 ± 160.69	99.60 ± 32.17
REN	4.15 ± 0.318	4.15 ± 0.60
UMPS	4.35 ± 0.318	4.30 ± 0.63

**Table 6:** Biological functions associated to the network of genes, from GeneMANIA^33^ analysis.

Function	False Discovery Rate
nucleobase metabolic process	3.02e-7
purine nucleobase metabolic process	8.22e-7
S-adenosylmethionine-dependent methyltransferase activity	2.70e-5
purine-containing compound salvage	6.06e-5
methyltransferase activity	6.65e-5
transferase activity, transferring one-carbon groups	8.29e-5
cellular metabolic compound salvage	6.95e-4
purine-containing compound biosynthetic process	1.75e-3
oxidoreductase activity, acting on the CH-NH2 group of donors, oxygen as acceptor	2.46e-3
N-methyltransferase activity	2.87e-3
oxidoreductase activity, acting on the CH-NH2 group of donors	3.48e-3
transferase activity, transferring pentosyl groups	3.20e-2
pigment biosynthetic process	4.08e-2
protein homotetramerization	5.07e-2
oxidoreductase activity, acting on the CH-CH group of donors	5.67e-2
histone methyltransferase activity	7.38e-2
pigment metabolic process	8.08e-2
macromolecule methylation	9.22e-2
ribonucleoside monophosphate biosynthetic process	9.57e-2
