# Supplementary material for: A theoretical study on predicted protein targets of apple polyphenols and possible mechanisms of chemoprevention in colorectal cancer
Source: Sci Rep. 2016 Sep 2;6:32516. doi: 10.1038/srep32516 (PMC5009435; doi:10.1038/srep32516)
Supplement: Supplementary Information [file srep32516-s1.pdf]

A theoretical study on predicted protein targets of apple polyphenols and possible mechanisms of chemoprevention in colorectal cancer

Bernardina Scafuri [1,2], Anna Marabotti[1,2], Virginia Carbone[1], Paola Minasi[1], Serena Dotolo[1], Angelo Facchiano[1,*]

[1] CNR-ISA, National Research Council, Institute of Food Science, Avellino, Italy

[2] Department of Chemistry and Biology "A. Zambelli", University of Salerno, Fisciano (SA), Italy

SUPPLEMENTARY MATERIALS

Supplementary Table 1. List of antioxidants tested with the reverse docking approach.

Name	Pubchem compound ID (CID)	Molecular structure
Avicularin (quercetin-3-*O*-arabinofuranoside)	5490064	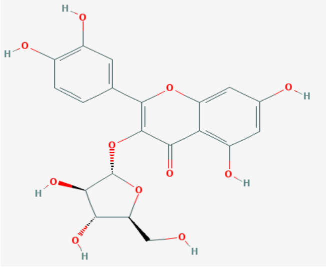
[+]-Catechin	9064	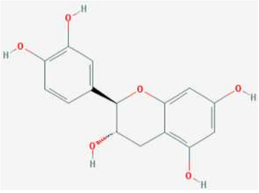
Chlorogenic acid	1794427	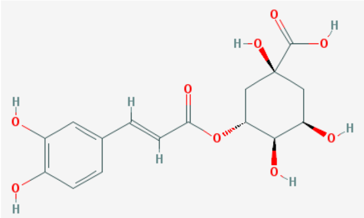
Cyanidin-3-galactoside	10299754	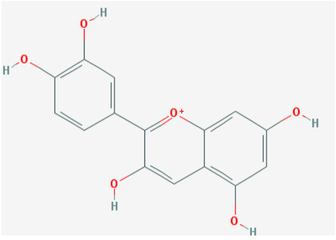
(-)-Epicatechin	72276	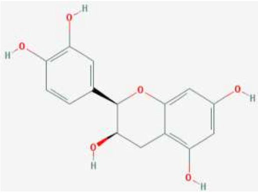

Hyperin (quercetin-3-*O*-galctoside)	5281643	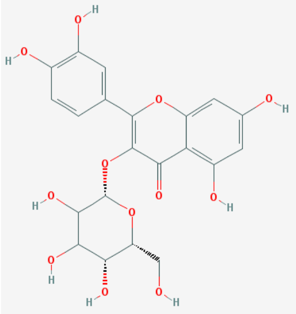
Isoquercitrin (quercetin-3-*O*-glucoside)	5280804	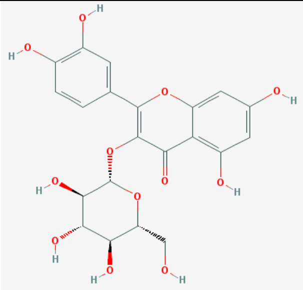
Phloridzin	6072	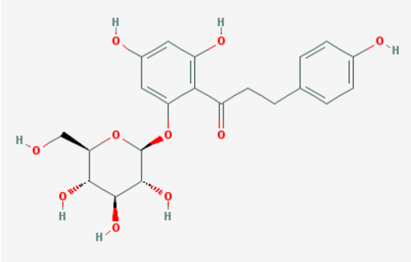
Procyanidin B_1	11250133	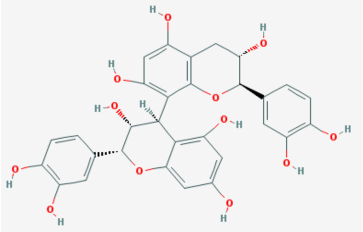
Procyanidin B_2	122738	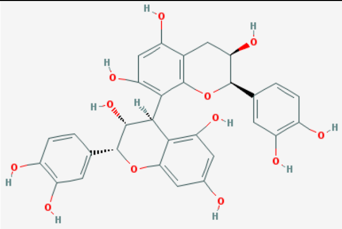
Quercetin	5280343	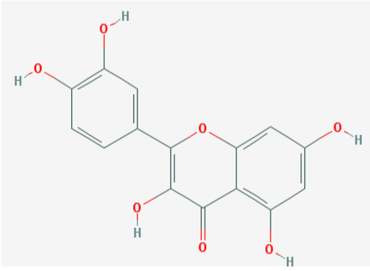

Quercitrin (quercetin-3-*O*-rhamnosid*e)*	5280459	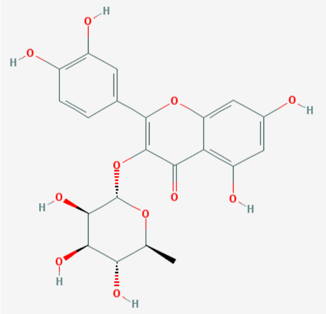
Reynoutrin (quercetin-3-*O*-xyloside)	5320863	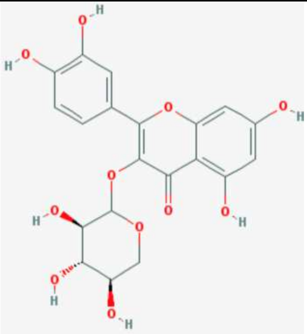
Rutin (quercetin-3-*O*-rutinoside)	5280805	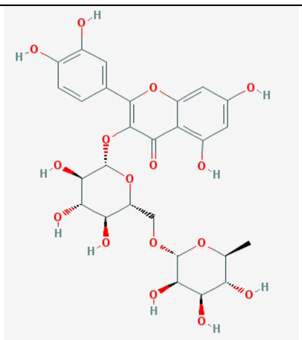

Supplementary Table 2: complete docking analysis on targets selected by idTarget for each antioxidant molecule. Results are presented for each ligand tested. For each protein-ligand couple, we show the best result in terms of energy, and the result with the most populated cluster of solutions, only if the difference in energy is not higher than 1.5 kcal/mol with respect to the best energetic result. When only one result is present, it represents both the best energetic and the most populated cluster of solutions.

[a]: the docking of cofactor was performed with a smaller box than the docking of ligand, because in the standard box the cofactor was not entering into the active site

[b]: water was left into the active site, otherwise the cofactor was not entering into the active site

[c]: PFM is the pyridoxal phosphate bound to the suicide inhibitor, it has been considered as a cofactor

[d]: This molecule is not classified as a cofactor in UniProt database (see Methods)

Ligand: AVICULARIN

PDB	Protein name	BLIND DOCKING	DOCKING ON BINDING SITE (with cofactors/ions)	DOCKING ON BINDING SITE (without cofactors/ions)	DOCKING OF COFACTOR
1BZY (chain A)	Hypoxanthine-guanine phosphoribosyltransferase	Representative run: 59<br>Binding energy: -6.55 kcal/mol<br>N. poses: 1<br>Binding site: YES	Not applicable	Representative run: 87<br>Binding energy: -11.06 kcal/mol<br>N. poses: 88	Not applicable
2QCG (chain A)	Uridine 5'-monophosphate synthase – orotidine-5'-monophosphate decarboxylase domain	**Result with best energy:**<br>Representative run: 56<br>Binding energy: -6.65 kcal/mol<br>N. poses: 10<br>Binding site: NO<br><br>**Result with most populated cluster at better energy:**<br>Representative run: 64<br>Binding energy: -6.57 kcal/mol<br>N. poses: 11<br>Binding site: NO	Not applicable	**Result with best energy:**<br>Representative run: 53<br>Binding energy: -7.17 kcal/mol<br>N. poses: 27<br><br>**Result with most populated cluster at better energy:**<br>Representative run: 16<br>Binding energy: -6.17 kcal/mol<br>N. poses: 28	Not applicable

Ligand: [+]-CATECHIN

PDB	Protein name	BLIND DOCKING	DOCKING ON BINDING SITE (with cofactors/ions)	DOCKING ON BINDING SITE (without cofactors/ions)	DOCKING OF COFACTOR
1C1Y (conformation A)	Ras related protein Rap 1A	**Result with best energy:**<br>Representative run: 85<br>Binding energy: -6.84 kcal/mol<br>N. poses: 5<br>Binding site: YES<br><br>**Result with most populated cluster at better energy:**<br>Representative run: 51<br>Binding energy: -5.90 kcal/mol<br>N. poses: 15<br>Binding site: NO	**Result with best energy:**<br>Representative run: 55<br>Binding energy: -6.33 kcal/mol<br>N. poses: 26<br><br>**Result with most populated cluster at better energy:**<br>Representative run: 12<br>Binding energy: -5.74 kcal/mol<br>N. poses: 29<br>**Binding site: PARTLY (only 1 residue)**	**Result with best energy:**<br>Representative run: 47<br>Binding energy: -8.49 kcal/mol<br>N. poses: 20<br><br>**Result with most populated cluster at better energy:**<br>Representative run: 78<br>Binding energy: -8.12 kcal/mol<br>N. poses: 58	Cofactor: GTP<br>Representative run: 30<br>Binding energy: -21.47 kcal/mol<br>N. poses: 27<br>Superposition with the cofactor in the crystallographic structure: YES
1C1Y (conformation B)	Ras related protein Rap 1A	**Result with best energy:**<br>Representative run: 62<br>Binding energy: -6.78 kcal/mol<br>N. poses: 2<br>Binding site:NO<br><br>**Result with most populated cluster at better energy:**<br>Representative run: 6<br>Binding energy: -5.54 kcal/mol<br>N. poses: 13<br>Binding site: NO	Representative run: 73<br>Binding energy: -6.48 kcal/mol<br>N. poses: 38	**Result with best energy:**<br>Representative run: 66<br>Binding energy: -8.48 kcal/mol<br>N. poses: 23<br><br>**Result with most populated cluster at better energy:**<br>Representative run: 89<br>Binding energy: -8.12 kcal/mol<br>N. poses: 55	Cofactor: GTP<br>**Result with best energy:**<br>Representative run: 24<br>Binding energy: -9.98 kcal/mol<br>N. poses: 13<br>Superposition with the cofactor in the crystallographic structure: YES<br><br>**Result with most populated cluster at better energy:**<br>Representative run: 59<br>Binding energy: -6.16 kcal/mol<br>N. poses: 26<br>Superposition with the cofactor in the crystallographic structure: **PARTLY**

2MMC<br>(Model 1)	GTP-binding nuclear protein Ran	**Result with best energy:**<br>Representative run: 66<br>Binding energy: -7.82 kcal/mol<br>N. poses: 13<br>Binding site: NO<br><br>**Result with most populated cluster at better energy:**<br>Representative run: 59<br>Binding energy: -6.26 kcal/mol<br>N. poses: 17<br>Binding site: NO	Not applicable	**Result with best energy:**<br>Representative run: 63<br>Binding energy: -5.49 kcal/mol<br>N. poses: 19<br><br>**Result with most populated cluster at better energy:**<br>Representative run: 58<br>Binding energy: -5.41 kcal/mol<br>N. poses: 40	Not applicable
3K8Y	GTP-ase Hras	**Result with best energy:**<br>Representative run: 30<br>Binding energy: -6.75 kcal/mol<br>N. poses: 4<br>Binding site: NO<br><br>**Result with most populated cluster at better energy:**<br>Representative run: 76<br>Binding energy: -6.17 kcal/mol<br>N. poses: 28<br>Binding site: NO	Representative run: 40<br>Binding energy: -5.75 kcal/mol<br>N. poses: 93	**Result with best energy:**<br>Representative run: 39<br>Binding energy: -8.87 kcal/mol<br>N. poses: 23<br><br>**Result with most populated cluster at better energy:**<br>Representative run: 90<br>Binding energy: -8.65 kcal/mol<br>N. poses: 75	Cofactor: GNP<br>**Result with best energy:**<br>Representative run: 86<br>Binding energy: -12.84 kcal/mol<br>N. poses: 9<br>Superposition with the cofactor in the crystallographic structure: YES<br><br>**Result with most populated cluster at better energy:**<br>Representative run: 94<br>Binding energy: -8.65 kcal/mol<br>N. poses: 14<br>Superposition with the cofactor in the crystallographic structure: **PARTLY**

4OBE (chain B)	GTP-ase Kras	Representative run: 70<br>Binding energy: -7.42 kcal/mol<br>N. poses: 20<br>Binding site: NO	**Result with best energy:**<br>Representative run: 12<br>Binding energy: -5.13 kcal/mol<br>N. poses: 23<br>**Binding site: NO**<br><br>**Result with most populated cluster at better energy:**<br>Representative run: 40<br>Binding energy: -4.96 kcal/mol<br>N. poses: 24<br>**Binding site: NO**	Representative run: 95<br>Binding energy: -7.72 kcal/mol<br>N. poses: 97	Cofactor: GDP[b]<br>Representative run: 91<br>Binding energy: -17.09 kcal/mol<br>N. poses: 40<br>Superposition with the cofactor in the crystallographic structure: YES

Ligand: CHLOROGENIC ACID

PDB	Protein name	BLIND DOCKING	DOCKING ON BINDING SITE (with co-factors/ions)	DOCKING ON BINDING SITE (without co-factors/ions)	DOCKING OF COFACTOR
1GRN	Protein Rho GTPase activing protein	**Result with best energy:**<br>Representative run: 94<br>Binding energy: -6.09 kcal/mol<br>N. poses: 1<br>Binding site: **PARTLY (only 2 residues)**<br><br>**Result with most populated cluster at better energy:**<br>Representative run: 100<br>Binding energy: -4.83 kcal/mol<br>N. poses: 5<br>Binding site: **NO**	**Result with best energy:**<br>Representative run: 66<br>Binding energy: -7.33 kcal/mol<br>N. poses: 7<br><br>**Result with most populated cluster at better energy:**<br>Representative run: 92<br>Binding energy: -5.71 kcal/mol<br>N. poses: 11<br>Binding site:	Representative run: 68<br>Binding energy:-8.49 kcal/mol<br>N. poses: 37	Cofactor: GNP<br>Representative run: 78<br>Binding energy:-12.04 kcal/mol<br>N. poses: 4
3K8Y	GTP-ase Hras	**Result with best energy:**<br>Representative run: 16<br>Binding energy: -11.65 kcal/mol<br>N. poses: 5<br>Binding site: NO<br><br>**Result with most populated cluster at better energy:**<br>Representative run: 59<br>Binding energy: -9.72 kcal/mol<br>N. poses: 9<br>Binding site: **PARTLY (only 1 residues)**	Representative run: 64<br>Binding energy: -6.36 kcal/mol<br>N. poses: 23	Representative run: 66<br>Binding energy:-10.56 kcal/mol<br>N. poses: 18	Cofactor: GNP<br>**Result with best energy:**<br>Representative run: 86<br>Binding energy: -12.84 kcal/mol<br>N. poses: 9<br><br>**Result with most populated cluster at better energy:**<br>Representative run: 94<br>Binding energy: -8.65 kcal/mol<br>N. poses: 14
3ZNN (chain A)	D-amino acid oxidase	Representative run: 23<br>Binding energy: -8.16kcal/mol<br>N. poses: 2<br>Binding site: YES	Representative run: 95<br>Binding energy: -9.23 kcal/mol<br>N. poses: 11	**Result with best energy:**<br>Representative run: 60<br>Binding energy: -7.11 kcal/mol<br>N. poses: 2<br>Binding site: NO<br><br>**Result with most populated cluster at better energy:**<br>Representative run: 91<br>Binding energy: -6.86 kcal/mol<br>N. poses: 24	Cofactor: FAD<br>Representative run: 32<br>Binding energy: -9.90 kcal/mol<br>N. poses: 2<br>Superposition with the cofactor in the crystallographic structure: **PARTLY**

4OBE (chain B)	GTP-ase Kras	Representative run: 35<br>Binding energy:-6.10 kcal/mol<br>N. poses: 2<br>Binding site: NO	**Result with best energy:**<br>Representative run: 93<br>Binding energy: -7.65 kcal/mol<br>N. poses: 27<br>**Binding site: YES**	Representative run: 93<br>Binding energy:-14.77 kcal/mol<br>N. poses: 84	Cofactor: GDP[b]<br>Representative run: 91<br>Binding energy: -17.09 kcal/mol<br>N. poses: 40<br>Superposition with the cofactor in the crystallographic structure: YES

Ligand: CYANIDIN-3-GALACTOSIDE

PDB	Protein name	BLIND DOCKING	DOCKING ON BINDING SITE (with cofactors/ions)	DOCKING ON BINDING SITE (without cofactors/ions)	DOCKING OF COFACTOR
1C1Y (Conformation A)	Ras Related protein Rap 1A	Representative run: 93<br>Binding energy: -5.53 kcal/mol<br>N. poses: 2<br>Binding site: NO	**Result with best energy:**<br>Representative run: 16<br>Binding energy: -5.89 kcal/mol<br>N. poses: 2<br><br>**Result with most populated cluster at better energy:**<br>Representative run: 55<br>Binding energy: -4.76 kcal/mol<br>N. poses: 34	Representative run: 64<br>Binding energy: -8.81 kcal/mol<br>N. poses: 70	Cofactor: GTP<br>Representative run: 30<br>Binding energy: -21.47 kcal/mol<br>N. poses: 27<br>Superposition with the cofactor in the crystallographic structure: YES
1C1Y (Conformation B)	Ras Related protein Rap 1A	Representative run: 47<br>Binding energy: -7.80 kcal/mol<br>N. poses: 3<br>Binding site: NO	**Result with best energy:**<br>Representative run: 53<br>Binding energy: -5.83 kcal/mol<br>N. poses: 3<br><br>**Result with most populated cluster at better energy:**<br>Representative run: 61<br>Binding energy: -4.68 kcal/mol<br>N. poses: 27	Representative run: 98<br>Binding energy: -9.09 kcal/mol<br>N. poses: 72	Cofactor: GTP<br>**Result with best energy:**<br>Representative run:24<br>Binding energy: -9.98 kcal/mol<br>N. poses: 13<br>Superposition with the cofactor in the crystallographic structure: YES<br><br>**Result with most populated cluster at better energy:**<br>Representative run: 59<br>Binding energy: -6.16 kcal/mol<br>N. poses: 26<br>Superposition with the cofactor in the crystallographic structure: **PARTLY**

1ZD9	ADP-ribosylation factor-like 10B	Representative run: 8<br>Binding energy: -5.48 kcal/mol<br>N. poses: 2<br>Binding site: NO	**Result with best energy:**<br>Representative run: 22<br>Binding energy: -5.88 kcal/mol<br>N. poses: 8<br><br>**Result with most populated cluster at better energy:**<br>Representative run: 90<br>Binding energy: -4.98 kcal/mol<br>N. poses: 14	**Result with best energy:**<br>Representative run: 35<br>Binding energy: -9.21 kcal/mol<br>N. poses: 4<br><br>**Result with most populated cluster at better energy:**<br>Representative run: 41<br>Binding energy: -7.81 kcal/mol<br>N. poses: 62	Cofactor: GTP<br>Representative run: 37<br>Binding energy: -17.93 kcal/mol<br>N. poses: 38<br>Superposition with the cofactor in the crystallographic structure: YES
2ICK	Isopentenyl diphophate delta isomerase	Representative run: 92<br>Binding energy: -6.18 kcal/mol<br>N. poses: 1<br>Binding site: NO	Representative run: 88<br>Binding energy: -4.90 kcal/mol<br>N. poses: 49	Representative run: 17<br>Binding energy: -4.96 kcal/mol<br>N. poses: 40	Not applicable (the only cofactor is Mg ion)
2MMC (Model 1)	GTP-binding nuclear protein Ran	Representative run: 97<br>Binding energy: -7.47 kcal/mol<br>N. poses: 4<br>Binding site: NO	Not applicable	**Result with best energy:**<br>Representative run: 96<br>Binding energy: -5,26 kcal/mol<br>N. poses: 2<br><br>**Result with most populated cluster at better energy:**<br>Representative run: 76<br>Binding energy: -4,99 kcal/mol<br>N. poses: 36	Not applicable
2RDU	Hydroxyacid oxidase 1	Representative run: 6<br>Binding energy: -5.52 kcal/mol<br>N. poses: 2<br>Binding site: NO	**Result with best energy:**<br>Representative run: 90<br>Binding energy: -7.05 kcal/mol<br>N. poses: 9<br>**Binding site: PARTLY (only 1 residue)**<br><br>**Result with most populated cluster at better energy:**<br>Representative run: 66<br>Binding energy: -4.49 kcal/mol<br>N. poses: 12<br>**Binding site: NO**	Representative run: 34<br>Binding energy: -9.07 kcal/mol<br>N. poses: 28	Cofactor: FMN<br>Representative run: 69<br>Binding energy: -13.57 kcal/mol<br>N. poses: 61<br>Superposition with the cofactor in the crystallographic structure: YES

4OBE (chain B)	GTP-ase Kras	Representative run: 2<br>Binding energy: -6.58 kcal/mol<br>N. poses: 3<br>Binding site: NO	Representative run: 35<br>Binding energy: -3.25 kcal/mol<br>N. poses: 8<br>**Binding site: NO**	**Result with best energy:**<br>Representative run: 96<br>Binding energy: -3.31 kcal/mol<br>N. poses: 4<br>**Binding site: NO**<br><br>**Result with most populated cluster at better energy:**<br>Representative run: 68<br>Binding energy: -3.12 kcal/mol<br>N. poses: 13<br>**Binding site: NO**	Cofactor: GDP[b]<br>Representative run: 91<br>Binding energy: -17.09 kcal/mol<br>N. poses: 40<br>Superposition with the cofactor in the crystallographic structure: YES

Ligand: [+]-EPICATECHIN

PDB	Protein name	BLIND DOCKING	DOCKING ON BINDING SITE (with cofactors/ions)	DOCKING ON BINDING SITE (without cofactors/ions)	DOCKING OF COFACTOR
1RM8	Matrix metalloproteinase 16	Representative run: 32<br>Binding energy: -10.37 kcal/mol<br>N. poses: 36<br>Binding site: YES	Not applicable	Representative run: 36<br>Binding energy: -10.58 kcal/mol<br>N. poses: 73	Not applicable
2C6Q (chainA)	GMP reductase 2	Result with best energy:<br>Representative run: 97<br>Binding energy: -7.97 kcal/mol<br>N. poses: 3<br>Binding site: YES<br><br>Result with most populated cluster at better energy:<br>Representative run: 37<br>Binding energy: -7.55 kcal/mol<br>N. poses: 6<br>Binding site: YES	Representative run: 30<br>Binding energy: -8.35 kcal/mol<br>N. poses: 63<br>Binding site: YES	Representative run: 90<br>Binding energy: -7.99 kcal/mol<br>N. poses: 50<br>**Binding site: PARTLY (only 3 residues)**	Cofactor: NADPH[d]<br>Representative run: 9<br>Binding energy: -6.56 kcal/mol<br>N. poses: 1<br>Superposition with the cofactor in the crystallographic structure: NO
2QCG (chain A)	Uridine 5'-monophosphate synthase – orotidine-5'-monophosphate decarboxylase domain	Result with best energy:<br>Representative run: 5<br>Binding energy: -8.15 kcal/mol<br>N. poses: 9<br>Binding site: YES<br><br>Result with most populated cluster at better energy:<br>Representative run: 11<br>Binding energy: -7.01 kcal/mol<br>N. poses: 17<br>Binding site: NO	Not applicable	Representative run: 1<br>Binding energy: -8.54 kcal/mol<br>N. poses: 57	Not applicable

2Z5F	Sulfotransferase family cytosolic 1B member 1	Representative run: 5<br>Binding energy: -6.77 kcal/mol<br>N. poses: 7<br>Binding site: NO	**Result with best energy:**<br>Representative run: 15<br>Binding energy: -5.53 kcal/mol<br>N. poses: 2<br><br>**Result with most populated cluster at better energy:**<br>Representative run: 21<br>Binding energy: -5.23 kcal/mol<br>N. poses: 48	Representative run: 13<br>Binding energy: -10.19 kcal/mol<br>N. poses: 78	Cofactor: ADP<br>Representative run: 41<br>Binding energy: -13.81 kcal/mol<br>N. poses: 29<br>Superposition with the cofactor in the crystallographic structure: YES

Ligand: HYPERIN

PDB	Protein name	BLIND DOCKING	DOCKING ON BINDING SITE (with cofactors/ions)	DOCKING ON BINDING SITE (without cofactors/ions)	DOCKING OF COFACTOR
2IW5	Lysine-specific histone demethylase 1	Representative run: 92<br>Binding energy: -2.11 kcal/mol<br>N. poses: 1<br>Binding site: NO	Representative run: 77<br>Binding energy: -6.01 kcal/mol<br>N. poses: 9	Representative run: 46<br>Binding energy: -9.06 kcal/mol<br>N. poses: 3	Cofactor: FAD[a]<br>Representative run: 74<br>Binding energy: -10.47 kcal/mol<br>N. poses: 7<br>Superposition with the cofactor in the crystallographic structure: YES
2OAT<br>(chain A)	Ornithine aminotransferase	Representative run: 21<br>Binding energy: -5.91 kcal/mol<br>N. poses: 1<br>Binding site: NO	Representative run: 50<br>Binding energy: -6.84 kcal/mol<br>N. poses: 19<br>**Binding site: PARTLY (only 2 residues)**	Representative run: 28<br>Binding energy: -7.39 kcal/mol<br>N. poses: 39	Cofactor: PFM<br>Representative run: 84<br>Binding energy: -9.03 kcal/mol<br>N. poses: 22<br>Superposition with the cofactor in the crystallographic structure: YES
3ORH	Guanidinoacetate N-methyltransferase	Representative run: 56<br>Binding energy: -5.52 kcal/mol<br>N. poses: 1<br>Binding site: NO	Not applicable	Representative run: 15<br>Binding energy: -10.30 kcal/mol<br>N. poses: 46	Not applicable

Ligand: ISOQUERCITRIN

PDB	Protein name	BLIND DOCKING	DOCKING ON BINDING SITE (with cofactors/ions)	DOCKING ON BINDING SITE (without cofactors/ions)	DOCKING OF COFACTOR
2OAT (chain A)	Ornithine aminotransferase	Representative run: 21<br>Binding energy: -7.62 kcal/mol<br>N. poses: 1<br>Binding site: PARTLY (Only 1 residue)	**Result with best energy:**<br>Representative run: 58<br>Binding energy: -6.97 kcal/mol<br>N. poses: 15<br>**Binding site: PARTLY (only 2 residues)**<br><br>**Result with most populated cluster at better energy:**<br>Representative run: 69<br>Binding energy: -5.65 kcal/mol<br>N. poses: 19	**Result with best energy:**<br>Representative run: 68<br>Binding energy: -7.68 kcal/mol<br>N. poses: 7<br><br>**Result with most populated cluster at better energy:**<br>Representative run: 78<br>Binding energy: -7.41 kcal/mol<br>N. poses: 14	Cofactor: PFM[c]<br>Representative run: 84<br>Binding energy: -9.03 kcal/mol<br>N. poses: 22<br>Superposition with the cofactor in the crystallographic structure: YES
3ORH	Guanidinoacetate N-methyltransferase	Representative run: 3<br>Binding energy: -6.07 kcal/mol<br>N. poses: 1<br>Binding site: NO	Not applicable	Representative run: 86<br>Binding energy: -10.88 kcal/mol<br>N. poses: 44	Not applicable

Ligand: PHLORIDZIN

PDB	Protein name	BLIND DOCKING	DOCKING ON BINDING SITE (with cofactors/ions)	DOCKING ON BINDING SITE (without cofactors/ions)	DOCKING OF COFACTOR
1ZRH	Heparan sulfate glucosamine 3-O-sulfotransferase 1	Representative run: 57<br>Binding energy: -4.81 kcal/mol<br>N. poses: 1<br>Binding site: NO	Not applicable	Representative run: 22<br>Binding energy: -10.27 kcal/mol<br>N. poses: 9	Not applicable
2X2E (chain D)	Dynamin-1	Representative run: 93<br>Binding energy: -4.00 kcal/mol<br>N. poses: 1<br>Binding site: NO	Representative run: 56<br>Binding energy: -6.52 kcal/mol<br>N. poses: 4<br>**Binding site: NO**	**Result with best energy:**<br>Representative run: 9<br>Binding energy: -9.62 kcal/mol<br>N. poses: 4<br><br>**Result with most populated cluster at better energy:**<br>Representative run: 18<br>Binding energy: -7.17 kcal/mol<br>N. poses: 16	Cofactor: GDP<br>**Result with best energy:**<br>Representative run: 43<br>Binding energy: -13.78 kcal/mol<br>N. poses: 6<br>Superposition with the cofactor in the crystallographic structure: YES<br><br>**Result with most populated cluster at better energy:**<br>Representative run: 91<br>Binding energy: -9.14 kcal/mol<br>N. poses: 49<br>Superposition with the cofactor in the crystallographic structure: **PARTLY**
3ATV	Heat shock 70 kDa protein 1A	Representative run: 21<br>Binding energy: -4.79 kcal/mol<br>N. poses: 2<br>Binding site: NO	Representative run: 37<br>Binding energy: -4.88 kcal/mol<br>N. poses: 11<br>**Binding site: PARTLY (only 2 residues)**	Representative run: 70<br>Binding energy: -6.99 kcal/mol<br>N. poses: 3	Cofactor: ADP<br>Representative run: 9<br>Binding energy: -4.59 kcal/mol<br>N. poses: 72<br>Superposition with the cofactor in the crystallographic structure: YES

3D3W	L-Xylulose Reductase	Representative run: 69<br>Binding energy: -5.69 kcal/mol<br>N. poses: 1<br>Binding site: NO	**Result with best energy:**<br>Representative run: 37<br>Binding energy: -6.79 kcal/mol<br>N. poses: 2<br><br>**Result with most populated cluster at better energy:**<br>Representative run: 25<br>Binding energy: -5.65 kcal/mol<br>N. poses: 10	Representative run: 78<br>Binding energy: -9.56 kcal/mol<br>N. poses: 1	Cofactor: NADP<br>Representative run: 78<br>Binding energy: -10.35 kcal/mol<br>N. poses: 1<br>Superposition with the cofactor in the crystallographic structure: YES
3ORH	Guanidinoacetate N-methyltransferase	Representative run: 73<br>Binding energy: -7.48 kcal/mol<br>N. poses: 1<br>Binding site: YES	Not applicable	Representative run: 71<br>Binding energy: -10.01 kcal/mol<br>N. poses: 13	Not applicable

Ligand: PROCYANIDIN B1

PDB	Protein name	BLIND DOCKING	DOCKING ON BINDING SITE (with cofactors/ions)	DOCKING ON BINDING SITE (without cofactors/ions)	DOCKING OF COFACTOR
2G1Y	Renin	Representative run: 30<br>Binding energy: -8.15 kcal/mol<br>N. poses: 1<br>Binding site: YES	Not applicable	**Result with best energy:**<br>Representative run: 90<br>Binding energy: -9.96 kcal/mol<br>N. poses: 18<br><br>**Result with most populated cluster at better energy:**<br>Representative run: 100<br>Binding energy: -9.49 kcal/mol<br>N. poses: 29	Not applicable
3KL6	Factor X, light chain	Representative run: 30<br>Binding energy: -7.27 kcal/mol<br>N. poses: 1<br>Binding site: NO	Not applicable	Representative run: 56<br>Binding energy: -3.08 kcal/mol<br>N. poses: 28<br>**Binding site: PARTLY (only 2 residues)**	Not applicable

Ligand: PROCYANIDIN B2

PDB	Protein name	BLIND DOCKING	DOCKING ON BINDING SITE (with cofactors/ions)	DOCKING ON BINDING SITE (without cofactors/ions)	DOCKING OF COFACTOR
3BUV	3-oxo-5-beta-steroid 4-dehydrogenase	Representative run: 84<br>Binding energy: -6.89 kcal/mol<br>N. poses: 13<br>Binding site: NO	Representative run: 49<br>Binding energy: -5.64 kcal/mol<br>N. poses: 51	Representative run: 8<br>Binding energy: -11.75 kcal/mol<br>N. poses: 48	Cofactor: NADP<br>Representative run: 5<br>Binding energy: -13.29 kcal/mol<br>N. poses: 3<br>Superposition with the cofactor in the crystallographic structure: YES
3FUN	Leukotriene A-4 hydrolase	Representative run: 79<br>Binding energy: -4.51 kcal/mol<br>N. poses: 2<br>Binding site: NO	**Result with best energy:**<br>Representative run: 10<br>Binding energy: -5.91 kcal/mol<br>N. poses: 4<br>**Binding site: PARTLY (only 1 residue)**<br><br>**Result with most populated cluster at better energy:**<br>Representative run: 42<br>Binding energy: -5.91 kcal/mol<br>N. poses: 14<br>**Binding site: PARTLY (only 1 residue)**	Representative run: 36<br>Binding energy: -8.70 kcal/mol<br>N. poses: 6	Not applicable (the only cofactor is the Zn ion)

Ligand: QUERCETIN

PDB	Protein name	BLIND DOCKING	DOCKING ON BINDING SITE (with cofactors/ions)	DOCKING ON BINDING SITE (without cofactors/ions)	DOCKING OF COFACTOR
1WBS	Mitogen-activated protein kinase 14	Representative run: 26<br>Binding energy: -6.56 kcal/mol<br>N. poses: 4<br>Binding site: YES	Not applicable	**Result with best energy:**<br>Representative run: 56<br>Binding energy: -7.15 kcal/mol<br>N. poses: 12<br>**Binding site: PARTLY (only 1 residues)**<br><br>**Result with most populated cluster at better energy:**<br>Representative run: 86<br>Binding energy: -7.15 kcal/mol<br>N. poses: 26	Not applicable
2QCG (chain A)	Uridine-5'-monophosphate synthase – orotidine-5'-monophosphate decarboxylase domain	**Result with best energy:**<br>Representative run: 40<br>Binding energy: -9.64 kcal/mol<br>N. poses: 9<br>Binding site: YES<br><br>**Result with most populated cluster at better energy:**<br>Representative run: 83<br>Binding energy: -6.90 kcal/mol<br>N. poses: 28<br>Binding site: NO	Not applicable	Representative run: 59<br>Binding energy: -9.75 kcal/mol<br>N. poses: 100	Not applicable
4LB4	Aldehyde reductase	Representative run: 2<br>Binding energy: -7.76 kcal/mol<br>N. poses: 45<br>Binding site: YES	Representative run: 10<br>Binding energy: -7.90 kcal/mol<br>N. poses: 100	**Result with best energy:**<br>Representative run: 4<br>Binding energy: -8.91 kcal/mol<br>N. poses: 14<br><br>**Result with most populated cluster at better energy:**<br>Representative run: 14<br>Binding energy: -7.71 kcal/mol<br>N. poses: 65	Cofactor: NADP[a]<br>Representative run: 1<br>Binding energy: -11.34 kcal/mol<br>N. poses: 5<br>Superposition with the cofactor in the crystallographic structure: YES

Ligand: QUERCITRIN

PDB	Protein name	BLIND DOCKING	DOCKING ON BINDING SITE (with cofactors/ions)	DOCKING ON BINDING SITE (without cofactors/ions)	DOCKING OF COFACTOR
1BZY (chain A)	Hypoxanthine-guanine phosphoribosyltransferase	Representative run: 77<br>Binding energy: -6.44 kcal/mol<br>N. poses: 8<br>Binding site: NO	Not applicable	Representative run: 40<br>Binding energy: -11.22 kcal/mol<br>N. poses: 70	Not applicable
2ZB4 (chain A)	Prostaglandin reductase 2	Representative run: 58<br>Binding energy: -6.82 kcal/mol<br>N. poses: 6<br>Binding site: NO	Representative run: 56<br>Binding energy: -7.29 kcal/mol<br>N. poses: 22	**Result with best energy:**<br>Representative run: 89<br>Binding energy: -10.46 kcal/mol<br>N. poses: 6<br><br>**Result with most populated cluster at better energy:**<br>Representative run: 71<br>Binding energy: -7.51 kcal/mol<br>N. poses: 19	Cofactor: NADP<br>Representative run: 67<br>Binding energy: -10.10 kcal/mol<br>N. poses: 2<br>Superposition with the cofactor in the crystallographic structure: YES

Ligand: REYNOUTRIN

PDB	Protein name	BLIND DOCKING	DOCKING ON BINDING SITE (with cofactors/ions)	DOCKING ON BINDING SITE (without cofactor and ions)	DOCKING OF COFACTOR
2OAT (chain A)	Ornithine aminotransferase	Representative run: 53<br>Binding energy: -7.48 kcal/mol<br>N. poses: 2<br>Binding site: YES	**Result with best energy:**<br>Representative run: 48<br>Binding energy: -6.74 kcal/mol<br>N. poses: 30<br><br>**Result with most populated cluster at better energy:**<br>Representative run: 75<br>Binding energy: -6.70 kcal/mol<br>N. poses: 40	Representative run: 34<br>Binding energy: -7.95 kcal/mol<br>N. poses: 70	Cofactor: PFM[c]<br>Representative run: 84<br>Binding energy: -9.03 kcal/mol<br>N. poses: 22<br>Superposition with the cofactor in the crystallographic structure: YES
2ZB4 (chain A)	Prostaglandin reductase 2	Representative run: 76<br>Binding energy: -7.67 kcal/mol<br>N. poses: 1<br>Binding site: YES	**Result with best energy:**<br>Representative run: 100<br>Binding energy: -7.37 kcal/mol<br>N. poses: 15<br><br>**Result with most populated cluster at better energy:**<br>Representative run: 44<br>Binding energy: -7.03 kcal/mol<br>N. poses: 32	Representative run: 51<br>Binding energy: -9.80 kcal/mol<br>N. poses: 10	Cofactor: NADP<br>Representative run: 67<br>Binding energy: -10.10 kcal/mol<br>N. poses: 2<br>Superposition with the cofactor in the crystallographic structure: YES

Ligand: RUTIN

PDB	Protein name	BLIND DOCKING	DOCKING ON BINDING SITE (with cofactors/ions)	DOCKING ON BINDING SITE (without cofactors/ions)	DOCKING OF COFACTOR
2BK3 (Chain A)	Amine Oxidase [Flavin-Containing] B	Representative run: 48<br>Binding energy: -4.88 kcal/mol<br>N. poses: 1<br>Binding site: NO	Representative run: 19<br>Binding energy: -6.47 kcal/mol<br>N. poses: 3<br>**Binding site: NO**	Representative run: 16<br>Binding energy: -8.38 kcal/mol<br>N. poses: 2	Cofactor: FAD[a]<br>Representative run: 2<br>Binding energy: -6.09 kcal/mol<br>N. poses: 6<br>Superposition with the cofactor in the crystallographic structure: YES
2FYT	Arginine N-methyltransferase 3	Representative run: 44<br>Binding energy: -4.60 kcal/mol<br>N. poses: 1<br>Binding site: NO	Not applicable	**Result with best energy:**<br>Representative run: 84<br>Binding energy: -7.91 kcal/mol<br>N. poses: 2<br><br>**Result with most populated cluster at better energy:**<br>Representative run: 33<br>Binding energy: -7.38 kcal/mol<br>N. poses: 9	Not applicable
2IW5	Lysine-specific histone demethylase 1	Representative run: 65<br>Binding energy: -2.41 kcal/mol<br>N. poses: 1<br>Binding site: NO	Representative run: 78<br>Binding energy: -6.29 kcal/mol<br>N. poses: 2	Representative run: 69<br>Binding energy: -5.91 kcal/mol<br>N. poses: 3	Cofactor: FAD[a]<br>Representative run: 74<br>Binding energy: -10.47 kcal/mol<br>N. poses: 7<br>Superposition with the cofactor in the crystallographic structure: YES
2OAT (chain A)	Ornithine aminotransferase	Representative run: 69<br>Binding energy: -5.16 kcal/mol<br>N. poses: 1<br>Binding site: NO	**Result with best energy:**<br>Representative run: 48<br>Binding energy: -4.84 kcal/mol<br>N. poses: 3<br>**Binding site: PARTLY (only 1 residue)**<br><br>**Result with most populated cluster at better energy:**<br>Representative run: 33<br>Binding energy: -4.83 kcal/mol<br>N. poses: 15	Representative run: 14<br>Binding energy: -8.81 kcal/mol<br>N. poses: 31	Cofactor: PFM[c]<br>Representative run: 84<br>Binding energy: -9.03 kcal/mol<br>N. poses: 22<br>Superposition with the cofactor in the crystallographic structure: YES

2ZB4 (chain A)	Prostaglandin reductase 2	Representative run: 63<br>Binding energy: -7.32 kcal/mol<br>N. poses: 1<br>Binding site: YES	**Result with best energy:**<br>Representative run: 24<br>Binding energy: -8.83 kcal/mol<br>N. poses: 7<br><br>**Result with most populated cluster at better energy:**<br>Representative run: 15<br>Binding energy: -7.97 kcal/mol<br>N. poses: 16<br>**Binding site: PARTLY (only 2 residues)**	Representative run: 27<br>Binding energy: -9.81 kcal/mol<br>N. poses: 2	Cofactor: NADP<br>Representative run: 67<br>Binding energy: -10.10 kcal/mol<br>N. poses: 2<br>Superposition with the cofactor in the crystallographic structure: YES
3ORH<br>(chain A)	Guanidinoacetate N-methyltransferase	Representative run: 94<br>Binding energy: -5.19 kcal/mol<br>N. poses: 1<br>Binding site: YES	Not applicable	Representative run: 88<br>Binding energy: -11.03 kcal/mol<br>N. poses: 63	Not applicable
